# Physiological Responses of Tomato Plants with Varied Susceptibility to Multiple Drought Stress

**DOI:** 10.3390/antiox14121448

**Published:** 2025-12-01

**Authors:** Hong Chen, Yi Liu, Fei Ding, Yankai Li, Carl-Otto Ottosen, Xiaoming Song, Fangling Jiang, Zhen Wu, Xiaqing Yu, Rong Zhou

**Affiliations:** 1 College of Horticulture, Nanjing Agricultural University, Nanjing 210095, China; 2023804316@stu.njau.edu.cn (H.C.); 2023104069@stu.njau.edu.cn (Y.L.); 2022204066@stu.njau.edu.cn (F.D.); 2023204044@stu.njau.edu.cn (Y.L.); ifl@njau.edu.cn (F.J.); zpzx@njau.edu.cn (Z.W.); 2 Department of Food Science, Aarhus University, Agro Food Park 48, N 8200 Aarhus, Denmark; coo@food.au.dk; 3 School of Life Sciences, North China University of Science and Technology, Tangshan 063000, China; songxm@ncst.edu.cn

**Keywords:** tomato, seedling stage, multiple drought, physiology, genotypic difference

## Abstract

Frequent extreme weather events exacerbate agricultural abiotic stress, with drought causing widespread yield loss. Tomato, a globally important vegetable sensitive to water deficit, has been predominantly studied under single-drought scenarios that poorly reflect recurrent field conditions. This study investigated physiological and molecular responses of two tomato genotypes to repeated drought stress. Results showed that the drought-sensitive genotype ‘TGTB’ exhibited faster ABA accumulation and more pronounced ABA-mediated stomatal closure. During the second drought cycle, stomatal pore length and width were significantly smaller than during the first drought, indicating a strong stress memory effect. In contrast, the drought-tolerant ‘LA1598’ showed minimal memory responses. Under extreme drought stress, primed and non-primed ‘TGTB’ plants showed significantly lower H_2_O_2_ content than controls, whereas primed ‘LA1598’ plants maintained a significantly lower O_2_^·−^ production rate than non-primed plants during both extreme drought cycles. Antioxidant enzyme systems contributed to ROS homeostasis, supported by the regulation of key drought-responsive genes. This study demonstrates genotype-dependent memory capacity and reveals that drought priming enhances repeated drought tolerance through ABA-regulated stomatal adjustment. These findings provide a theoretical basis for improving tomato resilience to recurrent drought and supporting breeding of drought-tolerant varieties.

## 1. Introduction

Tomatoes (*Solanum lycopersicum* L.) are an important vegetable crop worldwide, highly regarded for their flavor and nutritional value. According to 2020 data from the Food and Agriculture Organization of the United Nations (FAO), global annual production reached 183 million tons, with China accounting for over 64 million tons.

In the context of intensifying global climate change, drought has become the primary abiotic stressor threatening global agricultural production due to its wide reach, high frequency, and huge economic losses [1]. Geographic distribution data show that southern South America, the main body of the African continent, northeastern China, and North America are drought-prone regions [2]. Climate models predict that the proportion of global area subject to drought will dramatically expand from 15.4% to 44% by 2100, and drought risk index (DRI) for agricultural land will increase twofold from 52.45 to 104.60 by 2050 [3,4,5]. Droughts with varying severity consecutively occur over several years, alternating between mild and severe [6]. The impact of drought on food security exhibits a significant cascading amplification effect. From 1983 to 2009, approximately 454 million hectares of global arable land (75% of the harvested area) experienced yield reductions due to drought [7]. China, as a major agricultural producer and drought-prone region, has shown an increasing trend in drought occurrences from 1979 to 2018 [8,9]. Major food crops (wheat, maize, soybean, rice) and vegetables (cabbage, tomato, carrot) exhibit high sensitivity to drought stress, with their yield fluctuations directly impacting global food supply chain stability [10,11,12,13].

The biological mechanisms of plant response to drought are multidimensional and complex. At the phenotypic level, they are manifested in reduced plant height, decreased leaf area, reduced biomass, and dysregulated stomatal movement [14,15,16]. At the physiological metabolic level, reduced stomatal conductance leads to impaired CO_2_ assimilation, decreased photosynthetic rate, and disrupted transpiration efficiency, priming cascading effects [15,17,18]. At the biochemical level, altered Rubisco activity, increased proline content, elevated malondialdehyde accumulation, enhanced antioxidant enzyme levels, and activation of the ABA (abscisic acid) signaling pathway constitute typical stress responses [18,19,20,21]. At the molecular level, drought-induced expression of genes such as HmWRKY9 and SbNAC9 reveals plants’ epigenetic potential for stress adaptation [22,23].

In recent decades, most knowledge regarding drought tolerance mechanisms has been obtained by treating this stress as a single event occurring only once during a plant’s life cycle. This approach contrasts starkly with the frequent recurrence of drought events under natural conditions [24]. It is therefore imperative to find practical strategies to enhance plant resilience to repeated drought stress. Recent discovery of the phenomenon of “stress memory” has been recognized as a promising strategy for plants to cope with abiotic stresses under global change scenarios [25]. Plants pre-exposed to a specific stress can deploy enhanced or altered response strategies upon subsequent encounters with the same or other stresses [26]. The memory effect, developed after an initial stress exposure, significantly bolsters tolerance to subsequent stresses. This has been validated across diverse abiotic stresses and species, such as salt, heat, and drought stress in maize [27,28], and cold and heat stress in *Arabidopsis thaliana* [26,29,30,31], as well as cold stress in pepper [32]. In drought stress, photosynthetic efficiency and antioxidant enzyme activities of wheat were improved by drought pretreatment [33]. In potato, tuber dry matter translocation efficiency was improved by drought priming [34]. Drought priming also significantly improved drought stress response in soybean [35].

High water demand during its life cycle makes tomato highly vulnerable to drought stress [36], which has become a major constraint for tomato production. Although multiple stress response and stress memory is established in various plant species, critical knowledge gaps persist concerning the responses of tomato plants to repeated drought stress. As a model crop of significant basic research value and economic importance, in-depth analysis of these mechanisms in tomato is essential to guide the breeding of drought-tolerant varieties and the innovation of cultivation technologies.

This study aimed to investigate the dynamic physiological response patterns of tomato plants with different drought susceptibilities to periodic drought events. One drought-sensitive and one drought-tolerant genotype was first drought-primed, recovered, subjected to drought stress, and then recovered and subjected to drought stress again. Physiological indices as well as gene expression of tomato plants under different treatments were determined at different treatment time points. We hypothesized that the drought-sensitive genotype would exhibit more pronounced responses to periodic drought as compared with the drought-tolerant genotype at physiological and molecular levels. This study will refine the physiological mechanism of tomato in response to repeated drought stress and provide theoretical support for the development of new drought-resistant varieties.

## 2. Materials and Methods

### 2.1. Plant Materials

Drought-sensitive cultivated tomato ‘TGTB’ (Tomato Grape Telly Bean, *Solanum lycopersicum* L.) and drought-tolerant wild tomato ‘LA1598’ (*Solanum pimpinellifolium* L.) from Tomato Genetic Resource Center (TGRC, University of California, CA, USA) were used. Both ‘TGTB’ and ‘LA1598’ are indeterminate type with no significant difference in development. The seeds of the two genotypes were sown in 50-well trays (54 cm × 28 cm) filled with substrate, using a mixture of grass charcoal, perlite, and vermiculite (2:1:1 by volume). The trays were placed in a climate chamber of Nanjing Agricultural University (32°1′51 N, 118°50′25 E) at a temperature of 28 ± 5 °C with LED lamps (BN058C, ROYAL PHILIPS, Amsterdam, Noord-Holland, The Netherlands) to provide light. Daily day/night time was 15 h/9 h (7:00–22:00/22:00–7:00) with a light intensity of 15,000 lux in the chamber. Two weeks after sowing, the seedlings were irrigated every other day using 800 mL Stanley macronutrient water-soluble fertilizer (N + P_2_O_5_ + K_2_O ≥ 60%, nitrate nitrogen ≥ 5%, B 0.08%, EDTA-Zn 0.08%, EDTA-Fe 0.08%). The 20-day-old seedlings with three fully expanded leaves were transplanted into nutrient pots (top diameter 10.3 cm, bottom diameter 8 cm, height 8.5 cm) filled with the same substrate. During a 5-day acclimation period after transplanting, the same fertilizer was irrigated every other day, with 60 mL fertilizer per pot. The 25-day-old seedlings with four fully expanded leaves after acclimatization were subjected to the following treatments.

### 2.2. Experimental Design

**Figure 1:**
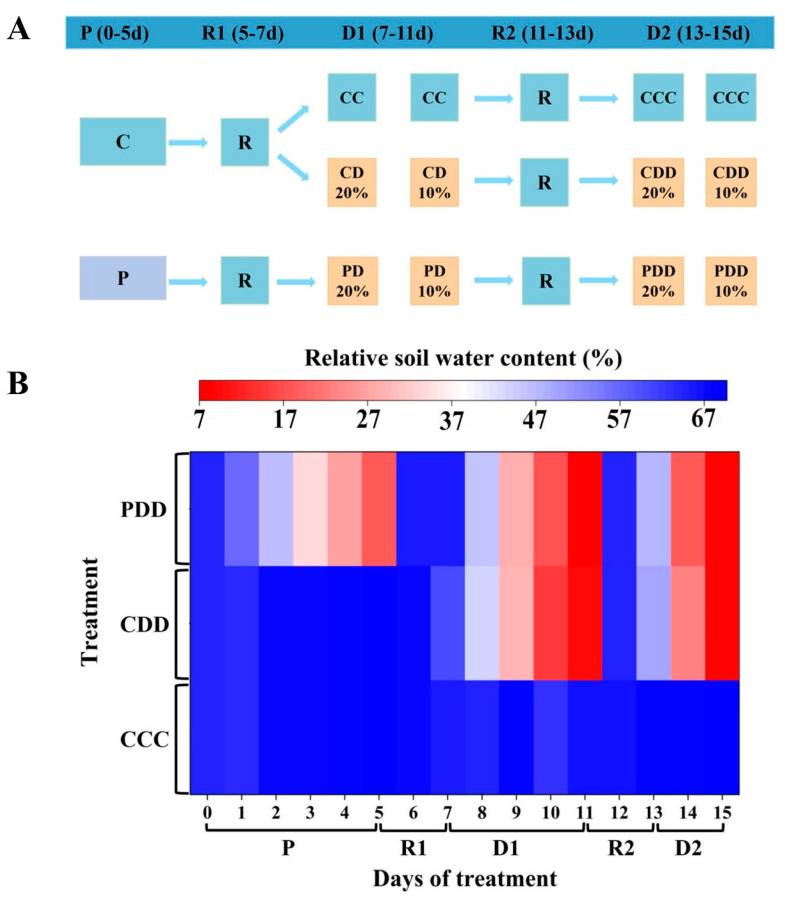
(**A**) Experimental design diagram and (**B**) relative soil water content (RSWC) during the treatment. Note: The treatments were divided into five stages, including the drought priming stage (P) (0–5 d), the first recovery (R1) (5–7 d), the first drought stress stage (D1) (7–11 d), the second recovery (R2) (11–13 d), and the second drought stress stage (D2) (13–15 d). During the P stage, C and P indicate control and priming. During the D1 stage, CC (control + control), CD (control + drought), and PD (priming + drought) indicate control, drought without priming and drought with priming, respectively, where 20% and 10% indicate average RSWC value. During the D2 stage, three treatments included CCC (control + control + control), CDD (control + drought + drought), and PDD (priming + drought + drought), where 20% and 10% indicate average RSWC value as well. CCC indicates control that was normally watered with 50 mL water per day and 60–80% RSWC. CDD indicates control followed by two drought treatments with 10–20% RSWC. PDD indicates drought priming with 20% RSWC followed by two drought treatments with 10–20% RSWC.

The treatments were divided into five stages, including the drought priming stage (P) (0–5 d), the first recovery (R1) (5–7 d), the first drought stress stage (D1) (7–11 d), the second recovery (R2) (11–13 d), and the second drought stress stage (D2) (13–15 d) (Figure 1A). Firstly, during the P1 stage from day 0 to day 5, C and P indicate control and priming, where priming was achieved by withholding water until soil relative water content (RSWC) reached 20%. Secondly, the pots were saturable irrigated by nutrient solution every day during the first recovery stage from day 5 to day 7, when the RSWC reached about 60%. Thirdly, during the D1 stage, CC (control + control), CD (control + drought), and PD (priming + drought) indicate control, drought without priming and drought with priming, respectively. Drought was achieved by withholding water until RSWC reached 10%. Fourthly, the second recovery stage followed the same procedure as the first recovery stage. Finally, during the D2 stage, three treatments included CCC (control + control + control), CDD (control + drought + drought), and PDD (priming + drought + drought), where 20% and 10% indicate average RSWC value as well. CCC indicates control that was normally watered with 50 mL water per day and 60–80% RSWC. CDD indicates control followed by two drought treatments with 10–20% RSWC. PDD indicates drought priming with 20% RSWC followed by two drought treatments with 10–20% RSWC. In general, the RSWC of drought-primed plants was approximately 20% during the P stage, while that of drought-stressed plants was approximately 10% during the D1 and D2 stage (Figure 1B). In total there were 120 plants, with 40 plants per treatment.

### 2.3. Measurements

The RSWC was monitored daily during the treatment period using a WET-2-K4 monitor (Delta-T, Cambridge, UK). The second- and third-node leaves of the plants (from top to bottom) provided the relevant measurements. Leaf samples were taken at P (day 5), D1-20% (day 10), D1-10% (day 11), D2-20% (day 14), and D2-10% (day 15) stages, to determine the ABA content, Rubisco activity, the production rate of superoxide anion (O_2_^·−^), hydrogen peroxide (H_2_O_2_) content, and antioxidant enzyme activity. Stomatal characteristics of abaxial leaves were measured at D1-10% (day 10) and D2-10% (day 11) stages. Expression of key genes in tomato plants responding to repeated drought stress were detected at P (day 5), R1 (day 7), D1-20% (day 10), D1-10% (day 11), R2 (day 13), D2-20% (day 14), and D2-10% (day 15) stages.

#### 2.3.1. ABA Concentration and Rubisco Activity

The ABA concentration and Rubisco activity were determined using the ELISA kit for plant ABA concentration (ML077235) and Rubisco activity (ML0027802) (Shanghai Enzyme-linked Biotechnology Co., Ltd., Shanghai, China). Firstly, the required strips were taken from the aluminum foil pouch after equilibrating at room temperature for 20 min. The 10 μL sample was added with 100 μL horseradish peroxidase (HRP)-labeled detection antibody, which was then incubated at 37 °C for 60 min. The liquid was discarded and the samples were added with 50 μL substrate A and B. The samples were then incubated at 37 °C for 15 min and mixed with 50 μL termination solution. The OD value was measured at 450 nm, and then the ABA concentration and Rubisco activity were calculated.

#### 2.3.2. Stomatal Characterization

Stomata characteristics were determined with reference to Zhou et al. (2020) using impression material (elite HD+, Zhermack, Badia Polesine, Italy) [20]. The abaxial leaves were imprinted, and the samples were observed and photographed using an orthogonal fluorescence microscope (DM6B, Leica, Wetzlar, Germany) at 63×. Three photographs were taken per replicate, and three stomata were selected per photograph. Length, width, and area of stomata and pore were measured using Image J 1.54f (NIH, Bethesda, MD, USA), and stomata number was counted.

#### 2.3.3. O_2_^·−^ Production Rate, H_2_O_2_ Content, and Antioxidant Enzyme Activities

The production rate of O_2_^·−^ was determined using the method described by Ke et al. (2007) [37]. A 0.2 g leaf sample was ground in liquid nitrogen, then mixed with 1.8 mL pre-chilled 50 mM PBS (pH 7.8) and centrifuged at low temperature. The 0.5 mL supernatant was mixed with 0.5 mL 50 mM phosphate-buffered saline (PBS) (pH 7.8) and 1 mL 10 mM hydroxylamine hydrochloride solution, then incubated at room temperature for 1 h. Subsequently, 1 mL 17 mM p-aminobenzenesulfonic acid and 1 mL 7 mM α-naphthylamine were added. After mixing, the samples were incubated at room temperature for 20 min. The absorbance was measured at 530 nm using a microplate reader (Cytation3, BioTek, Winooski, VT, USA) to calculate the O_2_^·−^ production rate of the leaves.

The H_2_O_2_ content was determined using the method described by Chakrabarty & Datta (2008) [38]. A 0.2 g leaf sample was ground in liquid nitrogen, then mixed with 1.8 mL l0.1% trichloroacetic acid (TCA) solution and centrifuged. The 0.5 mL supernatant was added to 2 mL 1 M KI solution and 0.5 mL 100 mM PBS (pH 7.0), followed by a 1 h dark reaction at room temperature. After the reaction, the absorbance was adjusted to zero with 0.1% TCA. The absorbance of the solution at 390 nm was measured using a microplate reader (Cytation3, BioTek, Winooski, VT, USA), and the H_2_O_2_ content in the sample was calculated.

The 0.2 g leaves were ground in liquid nitrogen and then mixed with 1.8 mL pre-chilled 50 mM PBS (pH 7.8). The samples were thoroughly mixed and centrifuged at 4 °C and 12,000× *g* for 20 min. The supernatant constituted the crude enzyme extract, which can be used to determine the activity of the following antioxidant enzymes.

Superoxide dismutase (SOD) activity was measured using the method of Zhou et al. (1997) [39]. The 40 µL crude enzyme extract was mixed with the 3 mL NBT reaction solution (50 mM PBS, pH 7.8 + methionine + EDTA-Na_2_ + riboflavin + NBT). The mixture was incubated at 25 °C under 20,000 Lux light for 20 min. After incubation, the optical density at 560 nm was measured using a microplate reader (Cytation3, BioTek, USA), and SOD activity was calculated. Peroxidase (POD) activity was measured using the method described by Muñoz-Muñoz et al. (2009) [40]. The 100 µL crude enzyme extract was mixed with 3 mL guaiacol-H_2_O_2_ reaction solution (200 mM PBS, pH 6.0 + guaiacol + H_2_O_2_), and the change in OD_470_ was measured within the first 120 s of the reaction. Catalase (CAT) activity was measured according to the method described by Aebi et al. (1984) [41]. A mixture of 0.1 mL crude enzyme extract and 2.9 mL H_2_O_2_ reaction solution (200 mM PBS, pH 7.8 + H_2_O_2_) was prepared, and the change in OD_240_ was measured within the first 120 s of the reaction. Soluble protein content was determined using Bradford method. Briefly, 100 µL of the crude enzyme extract was mixed with 2.9 mL Coomassie Brilliant Blue solution. Following a 2 min reaction period, protein concentration was measured at 595 nm using a microplate reader (Cytation 3, BioTek, USA). The activities of antioxidant enzymes including SOD, POD, and CAT were calculated on the basis of protein content.

#### 2.3.4. qRT-PCR

Total RNA of the samples was firstly extracted using the Plant Total RNA Extraction Reagent (AF504A, Pudi Biotech, Shanghai, China). The total RNA was then quality-checked using NanoPro 2010 (Dinghao Yuan Biotech, Tianjin, China), and samples that met the criteria of 1.9 < OD260/280 < 2.1, OD260/230 > 2.0, and total RNA > 1 µg of RNA samples were used for downstream experiments. The cDNA was generated by reaction using HiScript II 1st Strand cDNA Synthesis Kit (+gDNA wiper) kit (R212-01, Novozymes, Nanjing, China). The reaction of PCR program was 42 °C for 10 min, 50 °C for 15 min, and 85 °C for 2 min. The cDNA was generated using primer designing (https://www.ncbi.nlm.nih.gov, accessed on 14 April 2025) to design quantitative primers (Table 1). Actin from tomato was used as internal reference gene, and the qRT-PCR was performed using the TOROGreen^®^ qPCR Master Mix kit (No. QST-100P, TOROIVD, Shanghai, China). Reaction program was 95 °C for 60 s, 95 °C for 10 s, and 60 °C for 30 s, with a total of 40 cycles.

#### 2.3.5. Morphological Indicators

Plant height was measured from the growing point of the plant to the base of the stem using a ruler. Stem diameter was measured using vernier calipers. The above-ground part of the plant was cut off and weighed as fresh weight. The plant samples were put in an oven at 105 °C for 15 min and at 65 °C for 48 h until the weight was constant, which was then weighed as dry weight. Morphological indicators included nine biological replicates per treatment.

### 2.4. Data Analysis

Data were organized using Excel (Excel 2019, Redmond, WA, USA) and analyzed using IBM SPSS Statistics 27.0 (SPSS Inc., Chicago, IL, USA) (*p* < 0.05). Figures were made using Origin Pro 2024b (Origin Lab, Northampton, MA, USA).

## 3. Results

**Figure 2:**
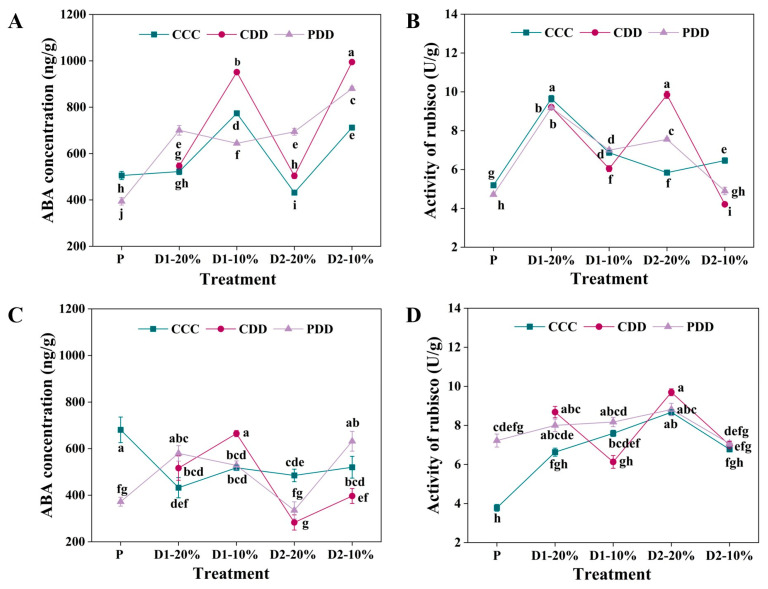
Abscisic acid (ABA) concentration and Ribulose-1,5-bisphosphate carboxylase/oxygenase (Rubisco) activity of (**A**,**B**) ‘TGTB’ and (**C**,**D**) ‘LA1598’. Note: P, D1-20%, D1-10%, D2-20%, and D2-10% correspond to Figure 1. P indicates priming stage (day 5). D1-20% and D1-10% indicate the first drought stress when the RSWC was approximately 20% and 10% (day 10 and day 11). D2-20% and D2-10% indicate the second drought stress when the RSWC was approximately 20% and 10% (day 14 and day 15). CCC (control + control + control) indicates control that was normally watered with 50 mL water per day and 60–80% RSWC. CDD (control + drought + drought) indicates control followed by two drought treatments with 10–20% RSWC. PDD (priming + drought + drought) indicates drought priming with 20% RSWC followed by two drought treatments with 10–20% RSWC. The data shown is mean ± SEM (*n* = 3). Different lowercase letters indicate significant differences (*p* < 0.05) among the treatments at the five stages.

For ‘TGTB’, the ABA concentration of PDD was significantly higher than that of CCC and CDD at 20% RSWC in both drought stress stages, i.e., D1-20% and D2-20% (Figure 2A). The ABA concentration of CDD was significantly higher than that of CCC and PDD at D1-10% and D2-10% (Figure 2A). For ‘LA1598’, there was no significant difference between the priming and non-priming treatments at 20% RSWC for both drought stresses (Figure 2C), that is, at the D1-20% and D2-20% stages. However, CDD exhibited the highest and lowest ABA concentration among the three treatments at D1-10% and D2-10%, respectively (Figure 2C).

There was no difference between the priming and non-priming Rubisco activities of genotype ‘TGTB’ at the D1-20% stage, but the CDD Rubisco activity was significantly lower than that of PDD at the D1-10% stage (Figure 2B). The CDD Rubisco activity was significantly higher than that of PDD at D2-20%, which significantly decreased and was lower than PDD at D2-10% (Figure 2B). Genotype ‘LA1598’ had significantly higher Rubisco activity than CCC at the initiation stage, i.e., the P stage (Figure 2D). The CDD Rubisco activity was significantly lower than PDD at D1-10%, and there was no difference among the CCC, CDD, and PDD treatments at D2 (Figure 2D).

**Figure 3:**
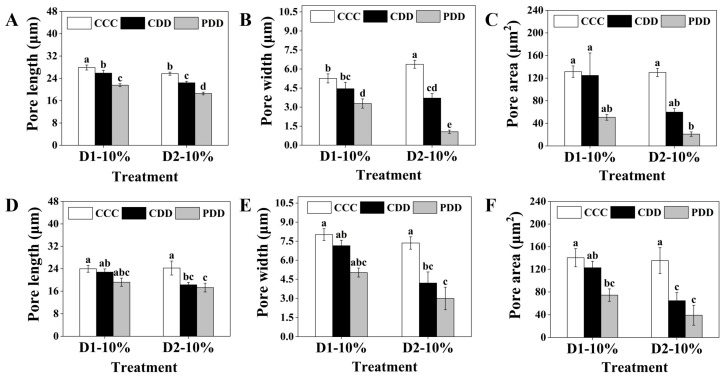
Pore length, width, and area of (**A**–**C**) ‘TGTB’ and (**D**–**F**) ‘LA1598’. Note: D1-10% and D2-10% correspond to Figure 1. D1-10% indicates the first drought stress when the RSWC was approximately 10% (day 11). D2-10% indicates the second drought stress when the RSWC was approximately 10% (day 15). CCC (control + control + control) indicates control that was normally watered with 50 mL water per day and 60–80% RSWC. CDD (control + drought + drought) indicates control followed by two drought treatments with 10–20% RSWC. PDD (priming + drought + drought) indicates drought priming with 20% RSWC followed by two drought treatments with 10–20% RSWC. The data shown is mean ± SEM (*n* = 27). Different lowercase letters indicate significant differences (*p* < 0.05) among the treatments at the five stages.

The pore length of PDD was significantly reduced compared to that of CDD at different stages of drought stress under the genotype ‘TGTB’ (Figure 3A). At D1-10%, the stomatal width of CDD was significantly higher than that of CCC (Figure S1B). For the stomatal width at the D1-10% stage, there was no significant difference between CDD and CCC, while PDD was significantly lower than both CCC and CDD. At the D2-10% stage, there were significant differences among treatments in CCC, CDD, and PDD. The stomatal width of CDD was significantly lower than that of CCC, while PDD was significantly lower than CDD. There were also significant differences between PDD treatments in the two drought stress stages (D1 and D2), with the stomatal width of PDD in the second drought stress stage being significantly smaller than that in the first stress stage (Figure 3B). At the D2-10% stage, the stomatal area induced by PDD was significantly smaller than that of CCC (Figure 3C). In the genotype ‘LA1598’, the stomatal length of PDD was significantly lower than that of CCC at the D2-10% stage (Figure S1E), and the stomatal width of both CDD and PDD was significantly lower than that of CCC at the D2-10% stage (Figure S1F). There was no significant difference in stomatal length between CDD and PDD at the D2-10% stage, but both were significantly lower than the control CCC (Figure 3D). Similarly, stomatal width at the D2-10% stage was significantly lower in CDD and PDD than in CCC (Figure 3E). For stomatal area, PDD was significantly lower than CCC at the D1-10% stage, while CDD and PDD were both significantly lower than CCC at the D2-10% stage (Figure 3F). In addition to stomatal characteristics, in terms of biomass accumulation, the plant height of CDD in ‘TGTB’ was significantly lower than CCC, while there was no significant difference between PDD and CCC (Figure S2A). There were no differences in dry weight among the CCC, CDD, and PDD treatments, but the fresh weight of CDD and PDD was significantly lower than that of CCC (Figure S2C). In ‘LA1598’, the plant height of CDD was also significantly lower than that of CCC (Figure S2D), and the fresh weight of CDD was significantly lower than that of CCC, while there was no significant difference in fresh weight between PDD and CCC (Figure S2F). The plant size and morphological changes of both tomato genotypes at different treatments shown in Figures S3 and S4 corresponded to the results of plant weight.

**Figure 4:**
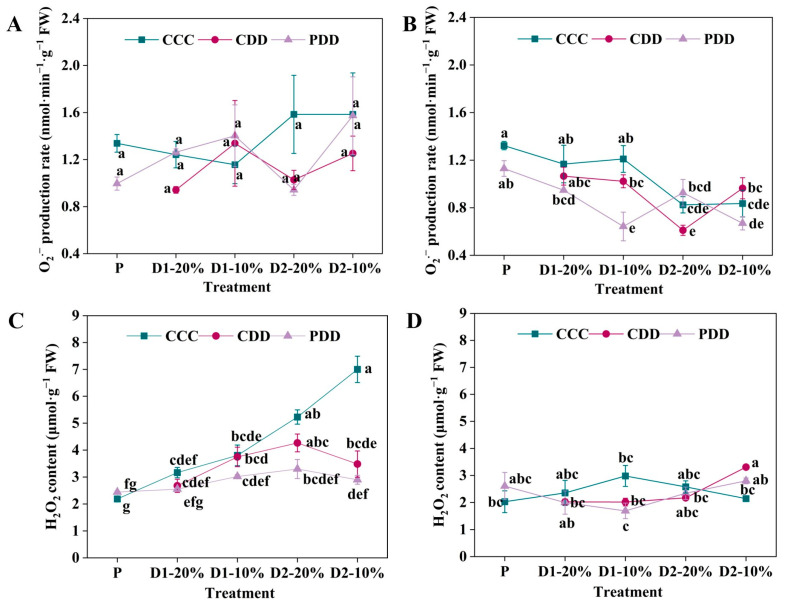
Hydrogen peroxide (H_2_O_2_) content and superoxide anion (O_2_^·−^) production rate of (**A**,**C**) ‘TGTB’ and (**B**,**D**) ‘LA1598’. Note: The treatment explanation is the same as Figure 2. The data shown is mean ± SEM (*n* = 3). Different lowercase letters indicate significant differences (*p* < 0.05) among the treatments at the five stages.

Even though there were no significant differences in O_2_^·−^ production rate of ‘TGTB’ under CCC, CDD, and PDD during different stages (Figure 4A), the H_2_O_2_ content of ‘TGTB’ was significantly lower under both CDD and PDD than CCC only at D2-10% (Figure 4C). By comparison, the O_2_^·−^ production rate of ‘LA1598’ significantly decreased under PDD as compared with CCC and CDD at D1-10% (Figure 4B). The O_2_^·−^ production rates of ‘LA1598’ were significantly lower under CDD than PDD at D2-20%, which was opposite at D2-10% (Figure 4B). Moreover, the H_2_O_2_ content of ‘LA1598’ significantly increased under CDD as compared with CCC at only D2-10% (Figure 4D).

**Figure 5:**
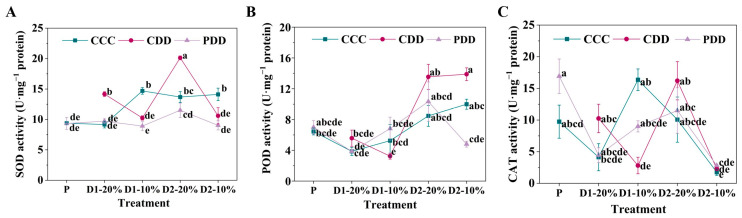
(**A**) Superoxide dismutase (SOD), (**B**) peroxidase (POD) activity and (**C**) catalase (CAT) activities of ‘TGTB’. Note: The treatment explanation is the same as Figure 2. The data shown is mean ± SEM (*n* = 3). Different lowercase letters indicate significant differences (*p* < 0.05) among the treatments at the five stages.

The SOD activity of ‘TGTB’ significantly increased under CDD as compared with CCC and PDD at D1-20% and D2-20%, which significantly decreased under CDD and PDD as compared with CCC at D1-10% and D2-10% (Figure 5A). By comparison, the POD activity of ‘TGTB’ was significantly lower under CDD than CCC at D1-10%, which was significantly higher at CDD than PDD at D2-10% (Figure 5B). The CAT activity of ‘TGTB’ significantly decreased under CDD as compared with CCC at D1-10% (Figure 5C).

**Figure 6:**
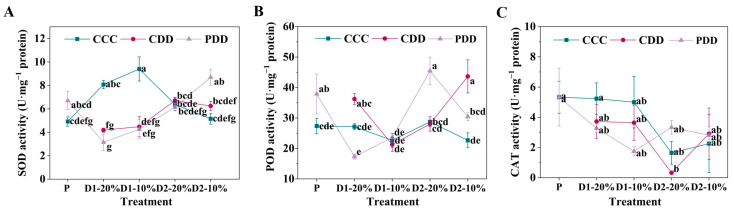
(**A**) Superoxide dismutase (SOD), (**B**) peroxidase (POD) activity and (**C**) catalase (CAT) activities of ‘LA1598’. Note: The treatment explanation is the same as Figure 2. The data shown is mean ± SEM (*n* = 3). Different lowercase letters indicate significant differences (*p* < 0.05) among the treatments at the five stages.

Furthermore, the SOD activity of ‘LA1598’ was significantly lower under CDD and PDD than CCC at D1-20% and D1-10%, which significantly increased under PDD as compared with CCC at D2-10% (Figure 6A). The POD activity of ‘LA1598’ was significantly higher under PDD than CCC at P, which significantly increased under CDD than PDD at D1-20% (Figure 6B). By comparison, the POD activity of ‘LA1598’ was significantly higher under PDD than CCC and CDD at D2-20%, which significantly increased under CDD than CCC and PDD at D2-10% (Figure 6B). However, no significant differences were observed in the CAT activity within different treatments at the same treatment stage (Figure 6C).

**Figure 7:**
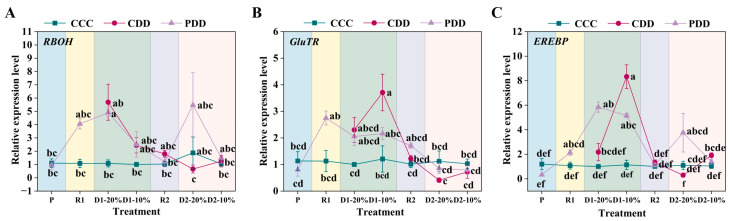
(**A**) Relative expression levels of *RBOH* (respiratory burst oxidase homolog), (**B**) *GluTR* (glutamate-tRNA reductase), and (**C**) *EREBP* (ethylene-responsive element binding proteins transcription factor) in ‘TGTB’. Note: P, R1, D1-20%, D1-10%, R2, D2-20%, and D2-10% correspond to Figure 1. P denotes the pre-treatment stage (day 5). R1 denotes the first recovery stage (day 7), while D1-20% and D1-10% represent the first drought stress, with RSWC approximately 20% and 10% (days 10 and 11). R2 denotes the second recovery phase, while D2-20% and D2-10% denote the second drought stress, with RSWC approximately 20% and 10% (days 14 and 15, respectively). CCC (control + control + control) indicates control that was normally watered with 50 mL water per day and 60–80% RSWC. CDD (control + drought + drought) indicates control followed by two drought treatments with 10–20% RSWC. PDD (priming + drought + drought) indicates drought priming with 20% RSWC followed by two drought treatments with 10–20% RSWC. The data shown is mean ± SEM (*n* = 3). Different lowercase letters indicate significant differences (*p* < 0.05) among the treatments at the five stages.

The relative expression of the *RBOH* (respiratory burst oxidase homolog) of genotype ‘TGTB’ at PDD was significantly higher than CCC at D1-20% (Figure 7A). The expression of *RBOH* at CDD showed a decreasing tendency from D1-20%, which was the least at the stage of D2-20% (Figure 7A). By comparison, the expression of *RBOH* at PDD decreased first but then increased and finally decreased during the treatment (Figure 7A). The relative expression of gene *GluTR* (glutamate-tRNA reductase) at PDD was significantly higher in the R1 stage than the P stage, which was significantly higher at CDD than CCC at D1-10% (Figure 7B). The relative expression of *EREBP* (ethylene-responsive element binding proteins transcription factor) in PDD was significantly higher in the R1 phase than in the P phase (Figure 7C). The expression of *EREBP* was significantly higher in PDD compared with the control at D1-20% (Figure 7C). Moreover, the expression of *EREBP* was significantly higher in PDD than CDD at D2-20% (Figure 7C).

**Figure 8:**
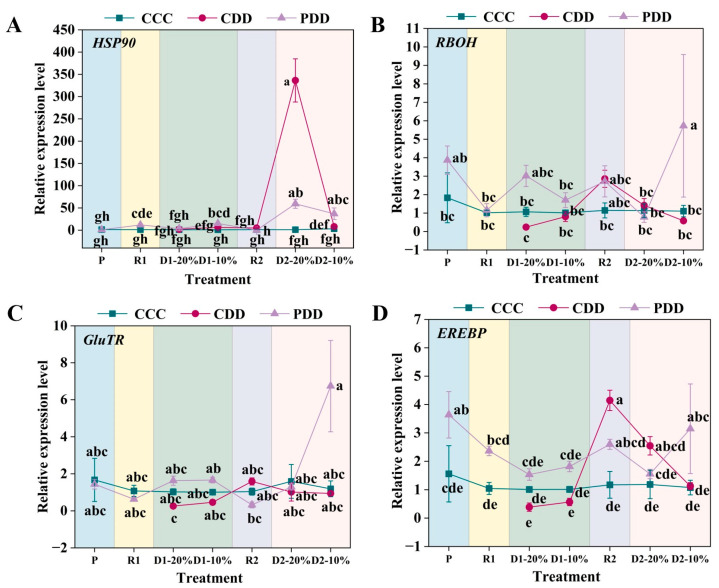
(**A**) Relative expression levels of *HSP90* (heat shock protein 90), (**B**) *RBOH* (respiratory burst oxidase homolog), (**C**) *GluTR* (glutamate-tRNA reductase), and (**D**) *EREBP* (ethylene-responsive element binding proteins transcription factor) in ‘LA1598’. Note: The treatment explanation is the same as Figure 7. The data shown is mean ± SEM (*n* = 3). Different lowercase letters indicate significant differences (*p* < 0.05) among the treatments at the five stages.

The expression of *HSP90* (heat shock protein 90) was significantly higher in PDD than CCC at R1, the relative expression of the PDD *HSP90* gene was significantly higher than that of the CCC and the CDD at the first drought soil moisture content 10% (D1-10%) stage, and there was no significant difference between the CDD and the CCC; at the D2-20% stage, PDD and CDD *HSP90* gene expression was significantly higher than that of CCC, and the relative expression of *HSP90* of the PDD gene was significantly higher than that of the CDD at the last second drought stress water content of 10% (D2-10%) of the treatment (Figure 8A). The relative expression of the *RBOH* gene of genotype ‘LA1598’ was significantly higher in PDD than in CCC and CDD at D2-10% (Figure 8B). The gene *GluTR* multiple treatment stages did not differ significantly among treatments, and the relative expression of the gene PDD increased only after the priming of the final stage of treatment, the D2-10% stage (Figure 8C). The relative expression of gene *EREBP* was significantly higher in the P period after PDD than in control, and the relative expression of gene *EREBP* was higher in treated PDD than in CCC and CDD at the final D2-10% stage of treatment (Figure 8D).

**Figure 9:**
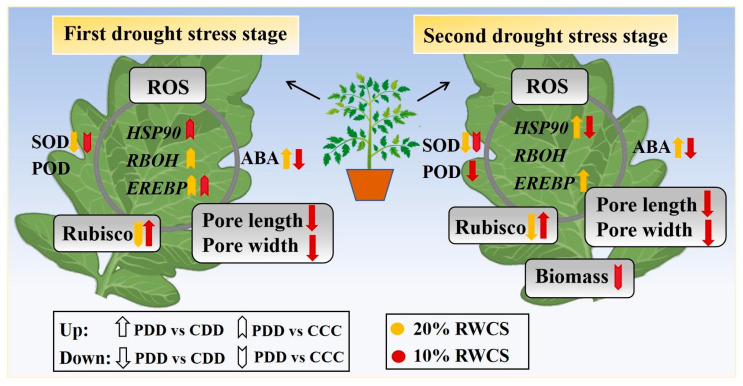
Physiological responses of tomato plants ‘TGTB’ to repeated drought stress. Note: CCC indicates control that was watered normally. CCC (control + control + control) indicates control that was normally watered with 50 mL water per day and 60–80% RSWC. CDD (control + drought + drought) indicates control followed by two drought treatments with 10–20% RSWC. PDD (priming + drought + drought) indicates drought priming with 20% RSWC followed by two drought treatments with 10–20% RSWC. RSWC: relative soil water content; ROS: reactive oxygen species; ABA: abscisic acid; SOD: superoxide dismutase; POD: peroxidase; *HSP90*: heat shock protein 90; *RBOH*: respiratory burst oxidase homolog; *EREBP*: ethylene-responsive element binding proteins transcription factor.

In genotype ‘TGTB’, ABA upregulated at D1-20% and D2-20%, but downregulated at D1-10% and D2-10% as a key signaling molecule (Figure 9). By acting on guard cells, the ABA fluctuation prompted stomatal closure in order to reduce water evaporation. Stomatal closure simultaneously impacted photosynthetic processes such as Rubisco, a key enzyme in photosynthesis, which was enhanced at both D1-10% and D2-10% (Figure 9). The efficient antioxidant enzyme system synergistically maintained ROS homeostasis in tomato plants under drought stress, during which key genes played roles as well (Figure 9).

## 4. Discussion

Years of research indicate that the effects of stress-induced memory and its mechanisms vary across species, genotypes, and stress duration [42,43,44,45]. The findings of this study provide compelling evidence: during the D1-20% and D2-20% stages of PDD treatment, ABA concentrations were significantly higher than in the CDD-treated group, suggesting that the ‘TGTB’ genotype exhibits a more rapid response to drought stress. However, this phenomenon was not observed in the ‘LA1598’ genotype (Figure 2). More importantly, the revealed genotypic differences extend beyond initial response speed to encompass the intensity and persistence of “memory.” Furthermore, after prior drought acclimation, ‘TGTB’ initiated synergistic defense networks—including ABA accumulation, stomatal closure, and dynamic regulation of antioxidant enzyme (Figure 3 and Figure 5)—earlier and more effectively during secondary stress, exhibiting classic “stress memory” characteristics. In contrast, the memory response in ‘LA1598’ was relatively delayed and transient, with significant stomatal regulation and enhanced Rubisco activity only observed during the first severe stress event (D1-10%) (Figure 2 and Figure 3). This indicates that ‘TGTB’ in both genotypes exhibits stronger stress memory capacity.

The memory mechanism involves the accumulation of signaling molecules that can be rapidly mobilized or activated during subsequent stress events. Taking ABA as an example, this key plant hormone responding to stresses like drought enhances drought tolerance by regulating morphophysiological and molecular processes [46,47,48]. In this study, when RSWC decreased to 20% and 10% in both drought stress events, ABA levels in pretreated ‘TGTB’ genotype plants were significantly higher than those in the control group and untreated plants. In contrast, ABA levels in untreated plants only rose sharply under 10% RSWC stress (Figure 2). This finding is consistent with previous studies: priming wheat exhibited elevated ABA levels in leaves during subsequent drought stress [49], and citrus plants also showed increased ABA levels after experiencing repeated drought stress [50]. Moreover, our research further reveals that priming enables ‘TGTB’ plants to rapidly activate the ABA synthesis pathway under milder stress (RSWC 20%), directly embodying the physiological manifestation of “memory”. ABA accumulation in drought-stressed plants regulates stomatal function through two mechanisms: (1) direct action on the stomatal complex; and (2) modulation of stomatal-related gene expression [48,51]. Here, ABA accumulation significantly reduced stomatal aperture in pretreated ‘TGTB’ plants during the D1-20% and D2-10% periods (Figure 3), which could minimize leaf water loss [52]. Thus, the rapid and enhanced ABA response constitutes the key physiological basis for the superior drought memory capacity of the ‘TGTB’ genotype. Additionally, drought-induced treatment upregulates Rubisco abundance during wheat grain filling [53]. We observed enhanced Rubisco activity in pretreated plants, occurring at the D1/D2-10% stage in the ‘TGTB’ genotype, but only at the D1-10% stage in the ‘LA1598’ genotype (Figure 2), further confirming the genotypic differences.

ROS, in forms of ^1^O_2_ (singlet oxygen), ·OH (hydroxyl radical), O_2_^·−^ and H_2_O_2_, play dual roles in plant stress responses, which was regarded as not only a key signal transduction molecule but also a toxic product when it was over-accumulated [54]. During the early stages of stress (from seconds to hours), plants rapidly generate ROS, with response characteristics varying among different cultivars [55,56]. Here, the O_2_^·−^ production rate showed no significant differences in ‘TGTB’ across treatment periods, while that was significantly higher in ‘LA1598’ under PDD than CDD at the D2-20% stage, which exhibited the opposite trend at the D2-10% stage (Figure 4). Excessive ROS can impair cellular functions, which has driven the evolution of a sophisticated antioxidant apparatus in plants to maintain ROS homeostasis and prevent oxidative damage [55,57]. The antioxidant enzyme system is involved in maintaining ROS homeostasis via altering the activities of antioxidant enzymes, during which the SOD eliminates O_2_^·−^ by catalyzing its dismutation, while POD and CAT are highly effective in degrading H_2_O_2_ [54,58]. The SOD constitutes the first line of defense in converting O_2_^·−^ to H_2_O_2_ [58]. Here, ‘TGTB’ primed plants showed stable SOD activity, but ‘LA1598’ primed plants exhibited increased SOD activity from D1-20% to D2-10% (Figure 5, Figure 6). By comparison, the POD activity of the two genotypes after priming revealed a similar trend with a peak at D2-20% (Figure 5 and Figure 6). Altogether, it was found that priming endows plants with memory, not only enabling faster stress perception (e.g., via ABA), but also facilitating more efficient preparation and activation of scavenging mechanisms to maintain redox homeostasis. This explains why ‘TGTB’ plants showed significantly lower H_2_O_2_ levels than the control at both D2-10% (Figure 4), indicating that the system had entered a more advantageous state of defense preparedness.

Beyond hormones, stomata, reactive oxygen species, and antioxidant enzyme systems, stress memory also involves transcriptional regulation of drought memory genes. *HSP90*, as a highly conserved multifunctional molecular chaperone, integrates environmental signals through interactions with co-chaperones to promote adaptive plant responses [59]. This gene family is widely recognized as a key factor enhancing plant heat and drought tolerance [60]. Xu et al. (2024) confirmed the crucial regulatory role of *HSP90* genes in drought stress responses of rose cultivars ‘Sweet Avalanche’ and ‘Wang Xifeng’ [61]. In ‘LA1598’, this phenomenon occurred at D1-10% and D2-10% (Figure 8A), indicating *HSP90*’s role in tomato responses to repeated drought stress. *RBOH*, as a key enzyme mediating H_2_O_2_ and O_2_^·−^ production, is a core enzyme in ROS generation and participates in plant responses to abiotic stress [62]. Studies indicate *RBOH* gene upregulation in poplar [63] and oats [64] under drought conditions. In this study, ‘TGTB’ exhibited significantly higher *RBOH* expression than CCC during D1-20% drought treatment (Figure 7A), while ‘LA1598’ showed significantly higher *RBOH* expression than CDD and CCC during D2-10% drought treatment (Figure 8B). *EREBP* participates in hormone and redox signaling, responding to stresses like drought and low temperature [65]. Its expression changes by 0.84-fold after 12 h drought treatment [66]. We found that ‘TGTB’ exhibited significantly higher *EREBP* expression than CCC at D1-20% under PDD conditions, and its expression was significantly enhanced compared to CDD at D2-20% (Figure 7C). Additionally, ‘LA1598’ exhibited significantly higher *EREBP* expression than CDD and CCC treatments during the D2-10% PDD phase (Figure 8D). Except for the *EREBP* gene in ‘LA1598’, expression levels of other genes during the priming showed no significant differences from the control group (Figure 7 and Figure 8). However, expression of these genes was generally elevated in pretreated plants during the R1 stage (Figure 7 and Figure 8), supporting that the theory that memory genes are activated during recovery [67]. Although stress memory is well documented in the literature, it can be reset (“forgotten”) through the process of growth recovery [26,67,68]. This reset may represent a trade-off strategy for plants, enabling them to benefit from past experiences while avoiding permanent fixation to previous stress states amid environmental uncertainty, thereby maximizing growth and reproductive potential. In this study, the more persistent gene expression activation in ‘TGTB’ (e.g., sustained high expression of *HSP90* at D2-20%) may precisely reflect its stronger memory capacity and slower reset.

## 5. Conclusions

In summary, the genotype ‘TGTB’ exhibited a pronounced stress memory effect following drought-induced treatment, specifically manifested by a more rapid ABA response during repeated drought. It effectively reduced water transpiration by regulating stomatal opening and closing (stomatal aperture at stage D2-10% was significantly smaller than at stage D1-10%), while maintaining high Rubisco activity (stages D1/D2-10%) and regulating antioxidant enzyme activity (SOD, POD, CAT), thereby effectively sustaining ROS homeostasis. In contrast, the ‘LA1598’ genotype exhibited weaker memory capacity, where the ABA response and stomatal regulation did not significantly increase during repeated drought stresses. Rubisco activity rose only during the first severe drought, indicating this genotype’s insensitivity to drought-induced memory effects. These results reveal significant differences in memory effects to repeated drought among tomato genotypes. The formation and maintenance of drought memory involve the expression regulation of genes such as *HSP90*, *RBOH*, and *EREBP*, whose expression patterns exhibit marked differences among genotypes. The physiological and molecular indicators identified in this study—including ABA dynamics, stomatal behavior, antioxidant enzyme activity, and expression patterns of genes like *HSP90* and *RBOH*—provide potential key trait markers for screening drought-tolerant tomato genotypes. Future research can build upon these findings to further elucidate the functions of these candidate genes and explore their applicability in marker-assisted breeding. Comprehensive analysis from perspectives including reactive oxygen species and plant hormone signaling, stomatal regulation, and photosynthetic capacity indicates that tomatoes exhibit both common and specific response patterns to repeated drought stress. The underlying mechanism may be related to resource trade-offs in biomass, suggesting that tomatoes adapt to periodic drought by regulating resource allocation between growth and stress resistance (Figure 9). This study systematically reveals the dynamic physiological response mechanisms of tomatoes under repeated drought stresses, providing crucial theoretical foundations for tomato cultivation and genetic improvement in regions with recurrent drought. Implementing drought priming during the seedling stage helps activate stress memory in strong memory genotypes like ‘TGTB’, thereby enhancing field tolerance of tomatoes in seasonal drought environments.

## Figures and Tables

**Table 1:** Primer sequence for qRT-PCR.

Gene Name	Gene ID	Forward Primer Sequence (5′-3′)	Reverse Primer Sequence (5′-3′)
*HSP90*	Solyc06g036290	GTACCTAAGAGGGCTCCATTTG	GTTCCTCACAGTTGTCCATGA
*RBOH*	Solyc03g117980	GTACGTCAGAAACTCGGTATGG	GACGCACTAAGGCCGATAAT
*GluTR*	Solyc01g106390	TCTCCAGCGGATCAGTATCA	CACACGAGCCATAGAAGAAGAA
*EREBP*	Solyc03g093550	GGAGATCCGTGACCCAAATAG	TTAAACGCTGCCCTGTCATA
*ACTIN*	LOC101264601	CTCTACATACTTGAGAGGTGCC	AGACGAGGAGAAAACATCACAA

Note: *HSP90* (heat shock protein 90), *RBOH* (respiratory burst oxidase homolog), *GluTR* (glutamate-tRNA reductase), and *EREBP* (ethylene-responsive element binding proteins transcription factor).

## Data Availability

The data underlying this article will be shared on reasonable request to the corresponding authors.

## Supplementary Materials

The following supporting information can be downloaded at: https://www.mdpi.com/article/10.3390/antiox14121448/s1. Figure S1. Stomatal length, width, area and number of (A–D) ‘TGTB’ and (E–H) ‘LA1598’ at D1-10% and D2-10%. Note: The D1-10% and D2-10% corresponded to Figure 1, indicating the relative soil water content (RSWC) was about 10% during the first and the second drought stress. CCC (control + control + control) indicates control that was normally watered with 50 mL water per day and 60–80% RSWC. CDD (control + drought + drought) indicates control followed by two drought treatments with 10–20% RSWC. PDD (priming + drought + drought) indicates drought priming with 20% RSWC followed by two drought treatments with 10–20% RSWC. Data are mean ± SE (*n* = 3). Different lower-case letters indicate significant differences (*p* < 0.05). Figure S2. Plant height, stem diameter, fresh and dry weight of (A–C) ‘TGTB’ and (D–F) ‘LA1598’ at D2-10%. Note: The D2-10% indicated the relative soil water content (RSWC) was about 10% during the second drought stress. CCC (control + control + control) indicates control that was normally watered with 50 mL water per day and 60–80% RSWC. CDD (control + drought + drought) indicates control followed by two drought treatments with 10–20% RSWC. PDD (priming + drought + drought) indicates drought priming with 20% RSWC followed by two drought treatments with 10–20% RSWC. Data are mean ± SE (*n* = 9). Different lower-case letters indicate significant differences (*p* < 0.05). Figure S3. Plant phenotypes of tomato genotype (A) ‘TGTB’ and (B) ‘LA1598’ at P, D1 and D2 stage. Note: The photos were taken at drought priming stage (P) on day 5, the first drought stress stage (D1) on day 11 d, and the second drought stress stage (D2) on day 15. CCC (control + control + control) indicates control that was normally watered with 50 mL water per day and 60–80% RSWC (relative soil water content). CDD (control + drought + drought) indicates control followed by two drought treatments with 10–20% RSWC. PDD (priming + drought + drought) indicates drought priming with 20% RSWC followed by two drought treatments with 10–20% RSWC.
