# Supplementary material for: Physiological Responses of Tomato Plants with Varied Susceptibility to Multiple Drought Stress
Source: Antioxidants (Basel). 2025 Dec 1;14(12):1448. doi: 10.3390/antiox14121448 (PMC12729441; doi:10.3390/antiox14121448)
Supplement: Supplementary file 1 [file antioxidants-14-01448-s001.zip › antioxidants-3905880-supplementary.pdf]

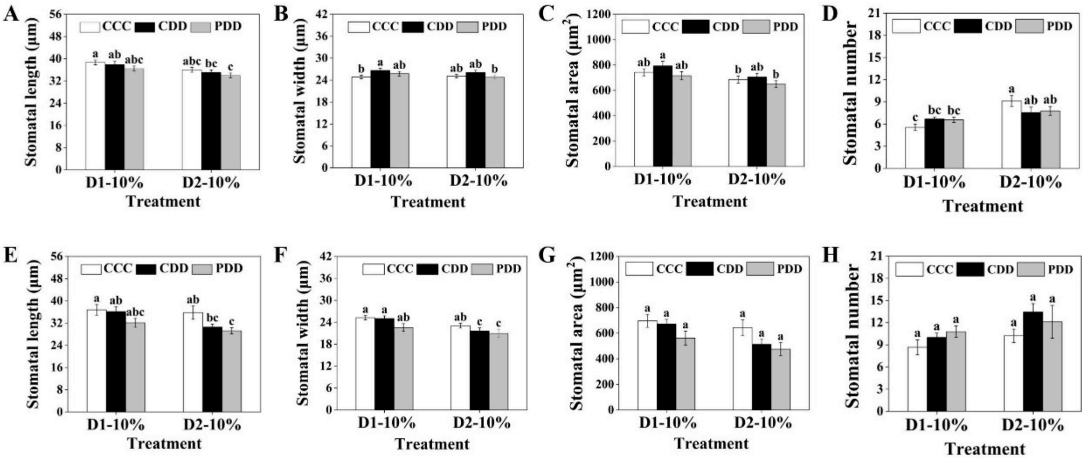

Figure S1. Stomatal length, width, area and number of **(A, B, C, D)** 'TGTB' and **(E, F, G, H)** 'LA1598' at D1-10% and D2-10%. Note: The D1-10% and D2-10% corresponded to Fig. 1, indicating the relative soil water content (RSWC) was about 10% during the first and the second drought stress. CCC (control + control + control) indicates control that was normally watered with 50 mL water per day and 60–80% RSWC. CDD (control + drought + drought) indicates control followed by two drought treatments with 10–20% RSWC. PDD (priming + drought + drought) indicates drought priming with 20% RSWC followed by two drought treatments with 10–20% RSWC. Data are mean ± SE (n = 3). Different lower-case letters indicate significant differences ($p < 0.05$).

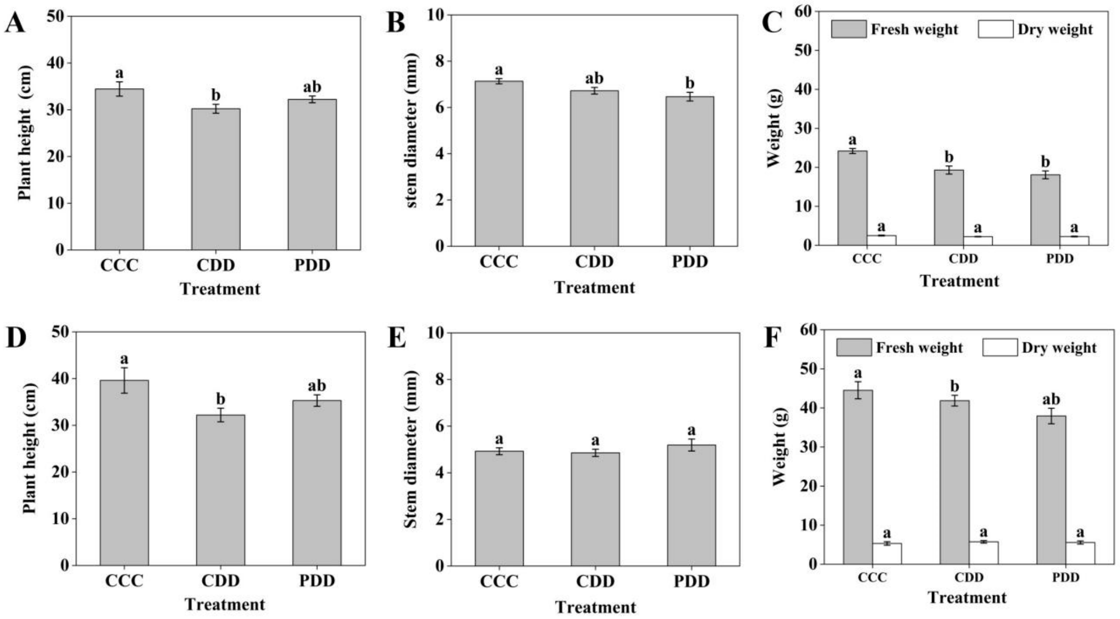

Figure S2. Plant height, stem diameter, fresh and dry weight of **(A, B, C)** 'TGTB' and **(D, E, F)** 'LA1598' at D2-10%. Note: The D2-10% indicated the relative soil water content (RSWC) was about 10% during the second drought stress. CCC (control + control + control) indicates control that was normally watered with 50 mL water per day and 60–80% RSWC. CDD (control + drought + drought) indicates control followed by two drought treatments with 10–20% RSWC. PDD (priming + drought + drought) indicates drought priming with 20% RSWC followed by two drought treatments with 10–20% RSWC. Data are mean ± SE (n = 9). Different lower-case letters indicate significant differences (p < 0.05).

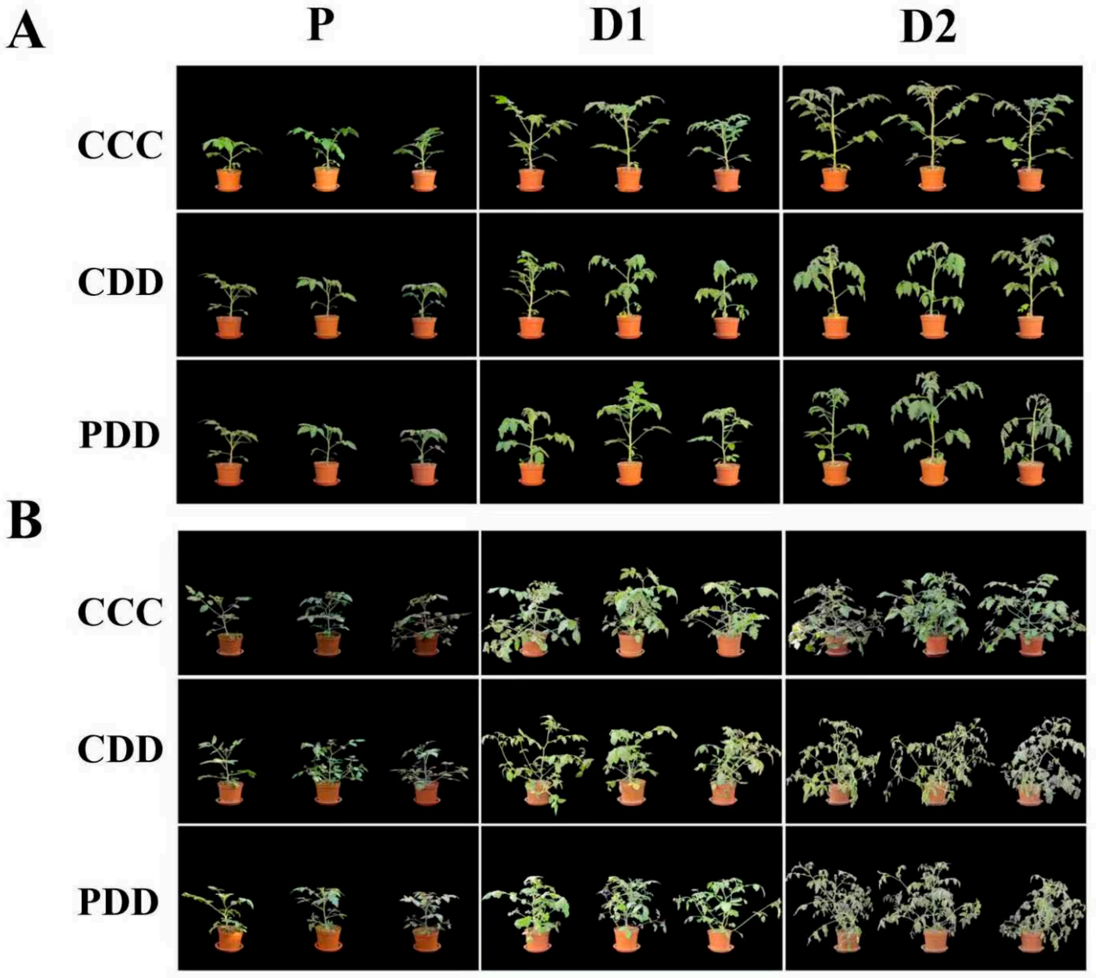

Figure S3. Plant phenotypes of tomato genotype **(A)** 'TGTB' and **(B)** 'LA1598' at P, D1 and D2 stage. Note: The photos were taken at drought priming stage (P) on day 5, the first drought stress stage (D1) on day 11 d, and the second drought stress stage (D2) on day 15. CCC (control + control + control) indicates control that was normally watered with 50 mL water per day and 60–80% RSWC (relative soil water content). CDD (control + drought + drought) indicates control followed by two drought treatments with 10–20% RSWC. PDD (priming + drought + drought) indicates drought priming with 20% RSWC followed by two drought treatments with 10–20% RSWC.
